# Impact of sulfamethoxazole, trimethoprim, diclofenac, carbamazepine, and their mixture on the metabolism of *Lemna minor*: a targeted metabonomic study

**DOI:** 10.1007/s11306-026-02405-9

**Published:** 2026-03-07

**Authors:** Rofida Wahman, Peter Schröder, Geoffroy Duporté, Serge Chiron, Jörg Drewes, Andrés Sauvêtre, Catarina Cruzeiro

**Affiliations:** 1 Chair of Urban Water Systems Engineering, Technical University of Munich, Am Coulombwall 3, 85748 Garching, Germany; 2 Present Address: Pharmacognosy Department, Faculty of Pharmacy, Assiut University, Assiut, Arab Republic of Egypt; 3 HydroSciences Montpellier, University of Montpellier, CNRS, IRD, Montpellier, France; 4 German Research Center for Environmental Health, Unit Environmental Simulation, Helmholtz Zentrum München, Ingolstädter Street 1, 85764 Neuherberg, Germany; 5 Present Address: Chair of Organic Agriculture and Agronomy, TUM School of Life Sciences Weihenstephan, Technical University of Munich, Liesel-Beckmann-Str. 2, 85354 Freising, Germany; 6 HSM, Univ Montpellier, IMT Mines Ales, CNRS, IRD, Ales, France; 7 Present Address: LSRE-LCM, ALiCE, Faculty of Engineering, University of Porto, Rua, Dr. Roberto Frias, 4200-465 Porto, Portugal

**Keywords:** Duckweed, Antibiotics, Anticonvulsant, LC-MS/MS, Analgesic, Focus orbitrap

## Abstract

**Introduction:**

Metabolomics is an analytical profiling technique that measures and compares large numbers of metabolites in biological samples, providing insight into metabolic mechanisms. There are few studies concerning the effects of xenobiotics and their transformation products on aquatic plant metabolites, which can uptake and detoxify them using untargeted metabolomics.

**Objectives:**

This study investigates how pharmaceuticals, including diclofenac (DCF) and carbamazepine (CBZ), as well as sulfamethoxazole (SMX) and trimethoprim (TRIM), present in aquatic environments, can influence the biosynthetic pathways of *Lemna minor*.Based on previous research on the effects of DCF, SMX, and TRIM on *Lemna* pathways, specifically phenylalanine, tyrosine, and tryptophan biosynthesis, folate biosynthesis, and the phenylpropanoid pathway, including flavonoid and anthocyanin metabolism.

**Methods:**

*Lemna* was incubated with DCF, CBZ, SMX, and TRIM alone and in a mixture (MIX) at 5 ppb (5 µg/L) for 5 days, at concentrations near environmental levels. The methanolic extract was analysed using a Q Exactive Focus Orbitrap to investigate changes in the aforementioned biosynthetic pathways, as reported in previous studies.

**Results:**

*Lemna* can modulate its pathways to produce more phenolic compounds as a defence mechanism against various drugs. This modulation can be considered an indicator for each drug.

**Conclusions:**

The presence of pharmaceuticals in the aquatic environment can affect the biosynthetic pathways of* Lemna*. Therefore,*Lemna minor* can be used as a model to study the stress-response of different pharmaceuticals on plant metabolites and their pathways.

**Graphical abstract:**

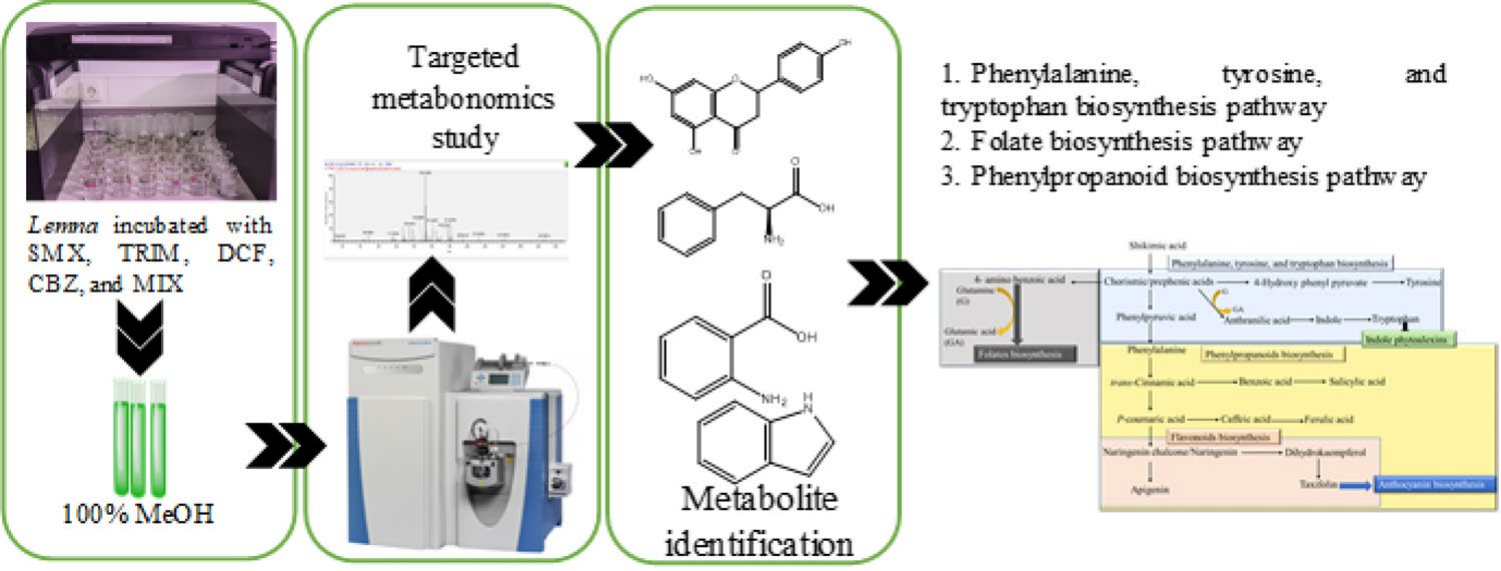

**Supplementary Information:**

The online version contains supplementary material available at 10.1007/s11306-026-02405-9.

## Introduction

Since the early 1940 s, the metabolomics approach has supported the idea that biological fluids reflect an individual’s health status. Metabonomics expands metabolic profiling by incorporating insights into metabolic disturbances induced by environmental factors. The distinction between “metabolomics” and “metabonomics” remains unclear; however, metabonomics is more commonly associated with NMR spectroscopy, while metabolomics is primarily linked to mass spectrometry-based techniques (Nicholson, 2006).

Plants contain various metabolites differing in molecular size, polarity, and functionality. Understanding plant natural products (plant metabolites) enables the development of new investigation strategies, which have matured as a valuable tool for advancing our understanding of plant biology and physiology. The basic goal of metabolomics is to provide a comprehensive qualitative and quantitative analysis of all metabolites in a living system. Interestingly, this concept could be extended to fingerprint investigations (Ernst et al., 2014; Heyman & Dubery, 2016; Sumner et al., 2015).

Pharmaceutical compounds (such as antibiotics: e.g., sulfamethazine, sulfamethoxazole (SMX), trimethoprim (TRIM), ciprofloxacin, and amoxicillin) can be absorbed and accumulated by plants at concentrations ranging from 6.9 to 48.1 µg·kg^−1^ (Zhang et al., 2017). For example, *Triticum aestivum* (common name: wheat) could uptake SMX, TRIM, ofloxacin, and carbamazepine (CBZ) when spray-irrigated with wastewater treatment plant (WWTP) effluent (Franklin et al., 2016). The impact of three commonly detected antibiotics in wastewater-ciprofloxacin HCl, oxytetracycline HCl, and sulfamethazine has also been studied in *Phragmites australis* at concentrations ranging from 0.1 to 1000 µg/L over 62 days. The study found that *Phragmites australis* could accumulate these antibiotics through passive absorption (Liu et al., 2013). Additionally, these antibiotics have been shown to exert toxic effects on leaf and root structures and chlorophyll composition at concentrations exceeding 10 µg/L in nutrient solutions over the same period (Liu et al., 2013). Incubation of *P. australis* for 4 days with diclofenac (10 and 100 µM) and carbamazepine (10 and 50 µM) alternated different pathways (Wahman et al., 2021).

The annual use of sulfonamides in veterinary medicine is approximately 5 times higher than total antibiotic use in some EU countries and South Korea, due to their broad-spectrum antimicrobial activity (Cheong et al., 2020). Sulfonamide antibiotics (e.g., SMX) resemble and substitute for p-aminobenzoic acid, which is involved in a wide variety of metabolic processes and possesses antioxidant, antimutagenic, protective, and reparative properties in *Lemna gibba* (Brain et al., 2008b). Thus, they are considered a competitive inhibitor of dihydropteroate synthetase, an enzyme involved in folate synthesis. Folate metabolism is involved in the biosynthesis of nucleic acids, proteins, lipids, and other biomolecules, as well as in epigenetic control via one-carbon unit transfer (Brain et al., 2008a). In 2019, Alkimin et al. reported that incubation of *L. minor* with diclofenac (DCF; 0, 4, 20, and 100 µg/L) decreased the content of photosynthetic pigments, the relative fluorescence decay values of chlorophyll, and the activities of oxidoreductases and dehydrogenases. However, it increased non-photochemical quenching, the amount of reactive nitrogen and oxygen species in roots, lipid peroxidation, oxidised ascorbate and thiols, and glutathione reductase activity. Further, our previous study showed alterations in *Lemna minor* phenylpropanoid pathways following incubation with DCF at 10 and 100 µM (3 and 29.6 µg/L) (Wahman et al., 2022). Anthropogenic activities provide a continuous input of CBZ into the environment (Clara et al., 2004), and this compound is not readily degraded in freshwater environments (Lajeunesse & Gagnon, 2007). Concentrations found in natural waters vary between 0.7 and 6.3 µg/L (Metcalfe et al., 2003; Ternes, 1998). It is an antiepileptic drug and a relatively lipophilic compound (*K*_ow_ = 2.2). Furthermore, incubating *L. minor* with CBZ, oxcarbazepine, acridine 9-carboxylic acid, and their mixture at environmental concentrations for 14 days altered nitrogen balance and chlorophyll indices. Besides, the phenolic compound index depended on pharmaceuticals and time of exposure, with no specific trend (Desbiolles et al., 2020).

The main concern in aquatic environments is the presence of pharmaceuticals (mainly antibiotics) that can affect the biosynthetic pathways of plants, such as *L. minor*, especially those that could be taken up and transformed by the plant.

Given the widespread presence and persistence of pharmaceuticals in aquatic environments, particularly antibiotics and psychoactive drugs, the central hypothesis of this study is that environmentally relevant concentrations of these compounds—individually and in mixture—can significantly disrupt key biosynthetic and stress-response pathways in *L. minor*.

Therefore, this work investigates the metabonomics of *L. minor* following exposure to diclofenac (DCF), carbamazepine (CBZ), sulfamethoxazole (SMX), trimethoprim (TRIM), and a mixture of these compounds at environmentally relevant concentrations. The aim is to characterise drug-specific and mixture-induced metabolic responses, with particular emphasis on pathways associated with plant stress adaptation and defence.

## Materials and methods

### Chemicals and reagents

Pesticide analytical-grade solvents (methanol for extraction) and LC/MS grade solvents (water, acetonitrile, formic acid 99%) were acquired from Carlo Erba (Val de Reuil, France). All solvents were filtered through a 0.22 μm nylon membrane filter (Nylaflo™, Michigan, USA) before use. Ultrapure water was generated by a Simplicity UV system (Millipore, Bedford, MA, USA) with a specific resistance of 18.2 MΩ cm at 25 °C. Analytically pure standards used for identification at level one (Sumner et al., 2007) were obtained from the following suppliers: Sigma-Aldrich (now part of Merck), Santa Cruz Biotechnology, Toronto Research Chemicals, and LGC Standards.

### Experimental design

*Lemna minor* (2 g fresh weight in 100 mL glass beakers) was exposed to SMX, TRIM, DCF, CBZ, and the mixture of them (MIX) at 5ppb (5 µg/L) in Steinberg media (constituents in Table S1) for 5 days (Fig. S1). Each exposure has five biological replicates. In addition, the solvent control (0.1% ethanol) was prepared in identical environmental conditions. After exposure, the plant material was rinsed with distilled water, gently dried on paper, and freeze-dried.

### Extraction method

Before extraction, the biological material was ground and homogenised under liquid nitrogen. *Samples* (42 mg) were then sonicated for 10 min at 4˚C with 35 kHz frequency (Heating Ultrasonic Water Bath, Advantage-Lab™ AL04-04, Thermo-Scientific, USA) in 1 mL of MeOH. Then, each extract was centrifuged at 1500 rpm/261.6 x g for 20 min at 4˚C (ST16R Refrigerated Centrifuge, Thermo Scientific, North Hampton, NH 03862, USA) and the supernatants were transferred to clean glass test tubes. The extraction process of biological replicates was triplicated in identical experimental conditions. Finally, the extracts were evaporated to dryness under nitrogen flux (N5.0, Air Liquide) and resuspended separately in 0.5 mL of 100% MeOH.

For protein precipitation, 10% of 5-sulfosalicylic acid was added to each extract, mixed, and centrifuged at 16,100 x g for 10 min at 4 °C (ST16R Refrigerated Centrifuge, Thermo Scientific, North Hampton, NH 03862, USA). The upper phase was collected and filtered using chromophil PTFE filters (25 mm, 0.22 μm pore size).

The samples were diluted 1:20 (v/v) in the mobile phase (H_2_O: ACN, 95:5% v/v), and an internal standard mixture (5 µL) was added before injection. The mixture consists of carbamazepine-d10, sulfamethoxazole-d4, diclofenac-d4, trimethoprim-d3, and ibuprofen, each at a final concentration of 50 ppb. This mixture was used to normalise the peak area of the corresponding sample.

### Instrument analysis

Samples were randomly separated with a reverse-phase discovery column (25 cm× 4.6 mm; 5 μm particle size; Sigma Aldrich). The mobile phase for the positive mode was water A, and acetonitrile as solvent B, both with 0.1% formic acid. For the negative mode, water (solvent C) and acetonitrile (solvent D) were used. The flow rate was 250 µL/min for both methods. The injection volume was 10 µL. Metabolite identification was performed using a Q Exactive Focus Orbitrap mass spectrometer (MS) (Thermo Fisher Scientific), equipped with a heated electrospray ionisation probe (HESI) source. The MS was operated at a mass resolution of 70,000 (FWHM, m/z 200) with a mass range of 50–600 m/z. The analyses in positive and negative ionisation modes were performed with 3.40 kV and 3.40 kV, 45 V and − 50 V, 90 V, and − 120 V, and 26 V and −25 V for spray, capillary, a tube lens, and skimmer voltages, respectively. The capillary temperature was 300 °C.

Samples were analysed in both positive and negative electrospray ionisation (ESI+) and (ESI-) modes.

### Quality control

For repeatable metabolic analyses, three features of the analytical system must be stable: retention time, signal intensity, and mass accuracy (Want et al., 2013). Therefore, quality control (QC) samples were prepared and injected at regular intervals during sample analysis to control analytical repeatability and sensitivity (Want et al., 2013). In addition, QCs were injected at the beginning of the analysis to evaluate column and metabolites’ performance within the same sample matrix. A pool of 10 µL of each biological sample (QC) was used to provide a true representation of the breadth of metabolites present in the sample set. Additionally, another QC sample was spiked with a mixture of target metabolites (50 ppb, each). The mixture consists of phenylalanine, tryptophan, indole, anthranilic acid, 

4-aminobenzoic acid, apigenin, glutamic acid, chorismic acid, prephenic acid, shikimic acid, *p*-coumaric acid,

*o*-coumaric acid, phenylpyruvate, tyrosine, benzoic acid, salicylic acid, caffeic acid, cinnamic acid, and 

4-hydroxyphenyl pyruvate. The pooled and spiked samples were used to determine the retention times (RTs) of the metabolites, along with system repeatability and sensitivity (Table 1).

**Table 1:** Targeted list of the *Lemna minor’s* metabolites modulated by the exposure to sulfamethoxazole, trimethoprim, diclofenac, carbamazepine, and a mixture of them at 5ppb (5 µg/L) in Steinberg media for 5 days. The molecular formula, calculated mass of the metabolites, mean, standard deviation, and relative standard deviation (RSD, %) of retention time (RT, min) in both quality control and experimental samples are presented. Additionally, the ionisation mode for each metabolite is provided

Name	Molecular Formula	Calculated mass (Da)	Quality control samples			Biological samples			Ionisation mode
			Mean RT (min)	±SD	RSD (%)	Mean RT (min)	ΔRT (min)	RSD of intensity (%)	
Anthranilic acid	C_7_H_7_NO_2_	137.0477	2.74	0.04	1.51	2.73	−0.01	25.3	+ve
4-Amino benzoic acid	C_7_H_7_NO_2_	137.0477	9.41	0.04	0.45	9.42	0.01	25.9	+ve
Phenylalanine	C_9_H_11_NO_2_	165.0790	5.58	0.04	0.67	5.59	0.01	10.3	+ve
Tryptophan	C_11_H_12_N_2_O_2_	204.0899	10.77	0.02	0.16	10.76	−0.01	4.8	+ve
Indole	C_8_H_7_N	117.0578	14.53	0.18	1.22	14.56	0.03	8.4	+ve
Glutamic acid	C_5_H_9_NO_4_	147.0532	1.66	0.00	0.28	1.65	−0.01	8.6	+ve
Apigenin	C_15_H_10_O_5_	270.0528	14.02	0.01	0.05	14.03	0.01	21.9	−ve
Chorismic/Prephenic acid	C_10_H_10_O_6_	226.0477	2.20	0.02	0.80	2.24	0.04	17.4	−ve
Shikimic acid	C_7_H_10_O_5_	174.0528	21.62	0.12	0.54	21.6	−0.02	2.6	−ve
Ferulic acid	C_10_H_10_O_4_	194.0579	8.65	0.12	1.43	8.61	−0.04	17.1	−ve
Dihydrokamepferol	C_15_H_12_O_6_	288.0634	13.24	0.02	0.16	13.25	0.01	12.2	−ve
Phenylpyruvate	C_9_H_8_O_3_	164.0473	8.39	0.02	0.26	8.40	0.01	4.8	−ve
*p*-Coumaric acid	C_9_H_8_O_3_	164.0473	2.24	0.03	1.12	2.23	−0.01	6.2	−ve
*O*-Coumaric acid	C_9_H_8_O_3_	164.0473	9.63	0.18	1.91	9.62	−0.01	14.5	−ve
Tyrosine	C_9_H_11_NO_3_	181.0739	4.77	0.08	1.59	4.80	0.03	6.5	−ve
Naringenin	C_15_H_12_O_5_	272.0685	14.15	0.03	0.19	14.17	0.02	9.8	−ve
Taxifolin	C_15_H_12_O_7_	304.0583	11.42	0.02	0.15	11.44	0.02	25.6	−ve
Benzoic acid	C_7_H_6_O_2_	122.0368	10.55	0.03	0.25	10.57	0.02	6	−ve
Salicylic acid	C_7_H_6_O_3_	138.0317	5.60	0.09	1.66	5.30	−0.30	20.8	−ve
4-Hydroxy-phenylpyruvate	C_9_H_8_O_4_	180.0423	1.89	0.03	1.43	1.92	0.03	13.6	−ve
Caffeic acid	C_9_H_8_O_4_	180.0423	8.00	0.12	1.55	7.95	−0.05	19.6	−ve
Cinnamic acid	C_9_H_8_O_2_	148.0524	12.09	0.16	1.35	12.14	0.05	28.3	−ve

*SD *standard deviation, *RT *retention time, *RSD* relative standard deviation

### Metabolomics identification

Data was acquired by Xcalibur software 4.0.27.19 (Thermo Fisher Scientific Inc., USA). The extracted ion chromatograms (EICs) were obtained from full-scan data-dependent MS2 (FMS-ddMS2) acquisition in Qual Browser software 4.0.27.19 (Thermo Fisher Scientific Inc., USA), using an inclusion list for each relevant compound m/z. Identification was based on comparisons of retention time, accurate mass, and MS2 fragments of the molecular ions listed in the inclusion list with those obtained from the standards.

A 5 ppm mass deviation was set. The signals of the compounds were divided by the signal of the respective deuterated carbamazepine-d10 standard in the positive mode, while the ibuprofen standard was used for the compounds in the negative mode. The carbamazepine-d10 standard was chosen because it had the most intense and stable signal of all internal standards. Relative signal intensities (relative peak area) were calculated by normalisation of the analyte/internal standard response ratio. Further data processing was conducted with Microsoft Excel 2016 (Washington, USA).

The mass absolute variation (Δ ppm) was less than 5 ppm. The RT standard deviation was less than 0.2. Moreover, the relative standard deviation (%RSD) was less than 1%, as shown in Table S2. These parameters were used to correlate the features in different samples.

The compounds were identified by comparing their masses, RTs, and fragment ions with those of the reference standards using Qual Browser software 4.0.27.19 (Thermo Fisher Scientific Inc., USA). The data were compared with the reference standard fragments (see Table 1) and the literature. They were identified by their RT and fragments when their masses matched. Compounds like *p*-coumaric acid, *o*-coumaric acid, and phenyl pyruvic acid had the same mass. The ratio of the two fragments, 116.9 and 119.1, was used to distinguish between *p*-coumaric acid and *o*-coumaric acid. Further, the phenyl pyruvic acid can be identified with the presence of the 117.3 fragment (peak) and the base ion peak at 119.1 (Fig. S3a). The naringenin and naringenin chalcone have the same mass and RT. They cannot be separated using the applied method; therefore, the peak area is calculated as the combined total of both naringenin and naringenin chalcone. A similar observation was made for chorismic acid and prephenic acid.

### Data and statistical analyses

Data were initially examined for normality using the Shapiro-Wilk test, and for homogeneity of variances using Levene’s test. To assess differences between treatments (DCF, CBZ, TRIM, SMX) and solvent control, a 1-way ANOVA was conducted, followed by the post-hoc Dunn’s multiple comparison test. The Unequal N HSD post hoc test was used for unbalanced data. A nonparametric Mann-Whitney U test was performed when normality or homogeneity of variances was not met. Statistical analyses were carried out using Statistica 7.0.

## Results and discussion

### Method performance regarding the selected metabolites

The metabonomics study of the phenylpropanoid pathway was conducted by isolating and identifying 22 metabolites (Table 1) after 5 days of exposure. This exposure period was selected to capture early metabolic responses to pharmaceutical stress, as *L. minor* is known to exhibit significant physiological and biochemical changes within a few days of treatment.

The metabolites were selected to enable the detection of a wide range of compounds using a single analytical method while minimising matrix effects from the extract. The selection of these metabolites was primarily influenced by the need to detect a broad range of compounds using a single analytical method. Their choice was mainly affected by detecting a large number of metabolites using the same method, as well as countering the matrix effect of the extract.

The LC–MS/MS system was calibrated as described in Sect. 2.5, resulting in an RT standard deviation below 0.2 min and a relative standard deviation (%RSD) of less than 2% (Table 1). Signal intensity repeatability was evaluated using QC samples, which showed a %RSD below 30%, indicating robust system performance even in a complex biological matrix (Want et al., 2013). These results confirm that the observed changes in signal intensity were attributable to incubation with the different drugs. Relative peak areas of individual metabolites were therefore calculated and compared with those of the control *Lemna* samples.

### The selection of metabolic pathways

**Fig. 1:**
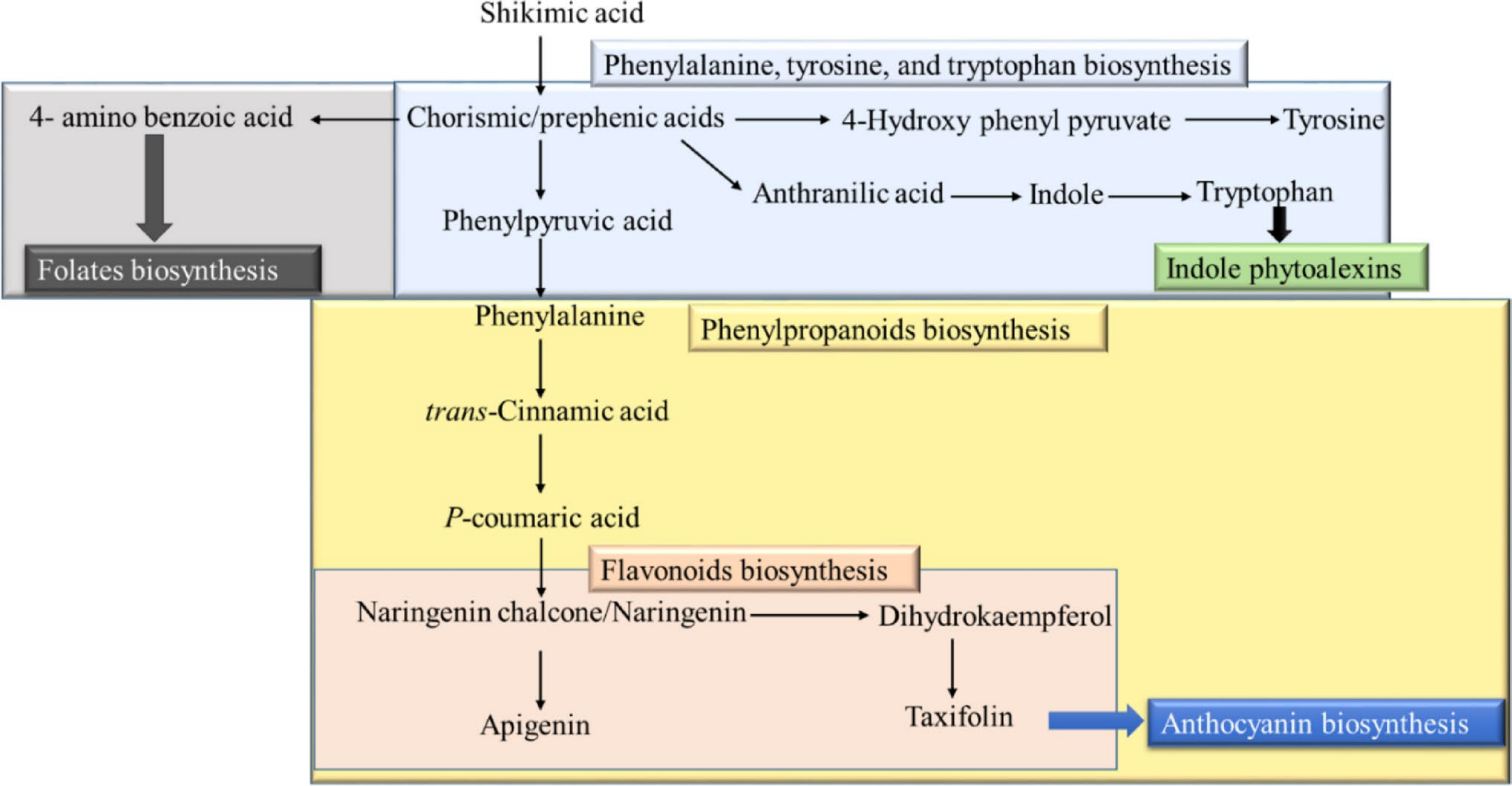
The schematic of the investigated biosynthetic pathway in *Lemna minor* based on the KEGG pathway database (https://www.genome.jp/kegg/pathway.html)

Previous studies showed that DCF and CBZ affect the flavonoids and anthocyanin pathways in *L. minor* and *Phragmites australis (*Wahman et al., 2022; Wahman et al., 2021). Also, SMX and TRIM affect the folate pathway (Brain et al., 2008b) (Fig. 1). Thus, the biosynthetic pathways of phenylalanine, tyrosine, tryptophan, folate, and phenylpropanoids (including flavonoid and anthocyanin pathways) were examined in *L. minor* samples exposed to DCF, CBZ, SMX, TRIM, and a combined mixture of these compounds at a concentration of 5 ppb.

Flavonoids are essential plant metabolites involved in defence, stress tolerance, and signalling. They are synthesised via the phenylpropanoid pathway, where p-coumaroyl-CoA and malonyl-CoA serve as key precursors, leading to the formation of naringenin—the central precursor of flavonoids and anthocyanins (Yonekura-Sakakibara et al., 2019). Upregulation of flavonoid biosynthesis is a common plant response to environmental stressors, including nutrient limitation, cold exposure, and nitrogen depletion (Olsen et al., 2009). Therefore, these pathways were selected as sensitive indicators of pharmaceutical-induced metabolic stress in *L. minor*.

### The effect of SMX, TRIM, DCF, CBZ, and MIX on the phenylalanine, tyrosine, and tryptophan pathways

**Fig. 2:**
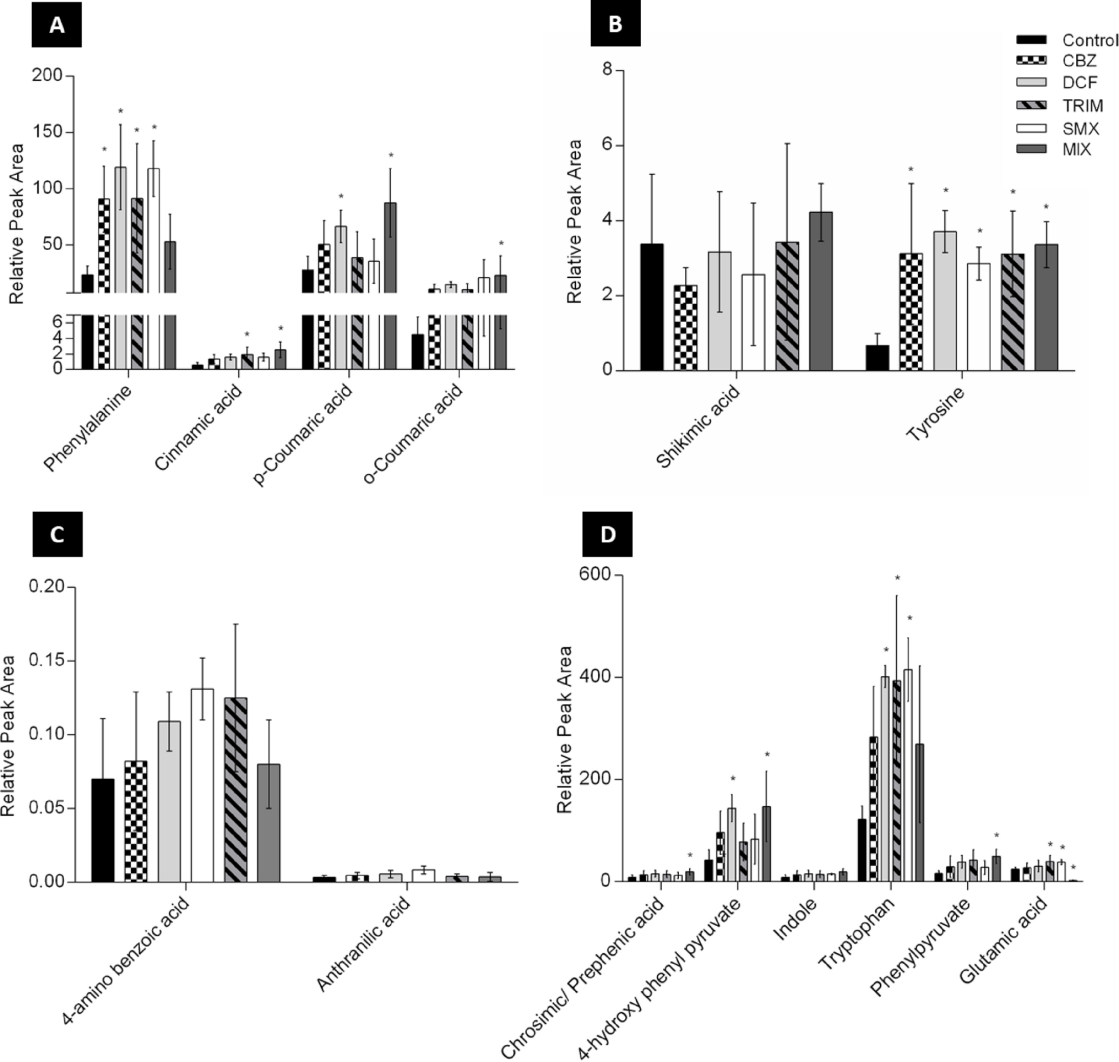
Relative peak area of metabolites of the phenylalanine, tyrosine, and tryptophan biosynthesis, folate biosynthesis, and phenylpropanoid pathways, identified in L. minor after 5 days of incubation with 5ppb of SMX, TRIM, DCF, CBZ, and their mixture, as well as the control group; statistical differences (p-value ≤ 0.05) against the respective control group are labelled with an asterisk “*”

Following SMX exposure, the precursor chorismic+prephenic acid (the sum of them) was up-modulated (1.4-fold) compared to controls (Fig. 2D). Chorismate is a key-branch point metabolite in the synthesis of all three aromatic acids, and secondary metabolites, such as folates (Tzin & Galili, 2010). Several intermediates were also increased, including phenylpyruvate (1.2-fold), 4-hydroxy phenylpyruvate (1.9-fold), anthranilic acid (2.4-fold), and indole (34-fold) (Fig. 2C and D), while shikimic acid showed a slight downregulation (Fig. 2B). This shift of metabolites suggests enhanced biosynthesis of various downstream compounds. Consistent with previous studies, inhibition of tryptophan feedback regulation can lead to accumulation of anthranilic acid (Brain et al., 2008a; Tzin & Galili, 2010). Furthermore, metabolites such as phenylalanine, tyrosine, and tryptophan biosynthesis are up-modulated (5-, 4.3-, and 4.3-fold, respectively) compared to the control (Fig. 2A, B and D). The up-modulation in phenylalanine in *Lemna minor* resulted in an up-regulation of the phenylpropanoid pathway and, hence, the flavonoid pathway.

TRIM exposure produced a similar response, significantly upregulating (*p*-value ≤ 0.05) the biosynthesis pathways of phenylalanine, tyrosine, and tryptophan compared to the control. Concentrations of precursor, intermediate, and final products increased as follows: chorismic/prephenic acid (1.7-fold), phenylpyruvate (1.5-fold), 

4-hydroxyphenylpyruvate (1.8-fold), anthranilic acid (1.2-fold), indole (4.1-fold), phenylalanine (3.9-fold), tyrosine (4.6-fold), and tryptophan (4-fold) (Fig. 2A-D).

DCF exposure showed that phenylalanine, tyrosine, and tryptophan biosynthesis pathways were also up-regulated (*p*-value ≤ 0.05). Chorismic/prephenic acid (1.8-fold) and tryptophan (4.1-fold) were up-modulated as in *Lemna* incubated with TRIM & SMX (Fig. 2D). Phenylpyruvate (2.4-fold) and anthranilic acid (2-fold) concentrations were lower than in TRIM treatment (Fig. 2C and D), whereas 4-Hydroxy phenylpyruvate (3.4-fold), indole (6.4-fold), phenylalanine (5.1-fold), and tyrosine (5.5-fold) were higher (Fig. 2A and D). Notably, indole concentration was higher in the SMX treatment than in the TRIM & DCF treatments, suggesting the presence of alternative indole-producing enzymes beyond tryptophan synthase. In maize, indole-3-glycerolphosphate lyase is responsible for forming the volatile indole under stress, and the tryptophan synthase-like enzyme (BX 1) produces natural benzoxazinoid pesticides 2,4-Dihydroxy-3,4-dihydro-2*H*−1,4-benzoxazin-3-one (DIBOA). Indole or benzoxazinoid production sometimes exceeds tryptophan (Buchanan et al., 2015). A similar mechanism may explain the reduced tryptophan levels observed at higher DCF concentrations (10 and 100 µM) and the previously reported detection of benzoxazinoid-related metabolites (Wahman et al., 2022).

CBZ exposure induced comparable responses to SMX, TRIM, and DCF treatment, although shikimic acid was down-modulated (1.2-fold), coinciding with a greater increase of phenylalanine than in SMX, TRIM, or DCF treatments (Fig. 2A). Indole increased (4.5-fold) above control extracts, while tryptophan was lower than in other treatments (SMX, TRIM, and DCF). Interestingly, 4-aminobenzoic acid accumulated in SMX and TRIM treatments, suggesting altered carbon flux and potential DNA-related stress responses under CBZ treatment (Fig. 2C).

In the mixture treatment (5 ppb each), shikimic acid appears to be upregulated (1.3-fold; Fig. 2B), and aromatic amino acid biosynthesis shows mild upregulation. Indole levels were lower than in single-compound exposures (Fig. 2D and Table S4), indicating enhanced channelling toward tryptophan synthesis.

Besides, tryptophan induces the production of phenylalanine and tyrosine. *Lemna minor* also increases the production of aromatic amino acids after incubation with glyphosate, metribuzin, or a mixture of both (Kostopoulou et al., 2020). Inhibition of 3-deoxy-D-arabinoheptulosonate-7-phosphate (DAHP) synthase enzyme by feedback loops is the usual way to regulate the amount of carbon in the pathway. SMX might be an antagonist to these three amino acids (phenylalanine, tyrosine, and tryptophan) in the feedback inhibition of DAHP synthase.

### The effect of SMX, TRIM, DCF, CBZ, and MIX on the folate pathway

SMX exposure tended towards increased levels (1.9-fold) of 4-aminobenzoic acid compared to control, consistent with earlier findings in *Lemna gibba* exposed to (10, 30, 100, 300, and 1000 µg/L) (Brain et al., 2008b). As sulfonamides inhibit dihydropteroate synthase, accumulation of upstream precursors is expected. Glutamic acid was also significantly upregulated (1.6-fold; *p*-value ≤ 0.05), indicating inhibition of folate biosynthesis in *L. minor*. Thus, SMX seems to block the folate biosynthesis pathway in *Lemna minor* as in bacteria.

TRIM produced a similar effect to SMX on bacteria, inhibiting folate biosynthesis at different positions along the pathway (see proposed pathway in Fig. S2). In this experiment, 4-amino benzoic acid (1.8-fold) and glutamic acid (1.7-fold, p-value ≤ 0.05) increased, reflecting compensatory responses to folate pathway inhibition at a different enzymatic step (Fig. 2C and D) (Brain et al., 2008a).

DCF induced a weaker accumulation of 4-aminobenzoic acid (1.6-fold) and glutamic acid (1.3-fold) when compared to SMX and TRIM treatments (Fig. 2C and D). This indicates that SMX and TRIM block the folate pathway in *Lemna.* Accumulation of 4-aminobenzoic acid was observed across all treatments, including CBZ and MIX, likely reflecting increased one-carbon metabolism and stress-related DNA responses (Shi et al., 2023).

**Fig. 3:**
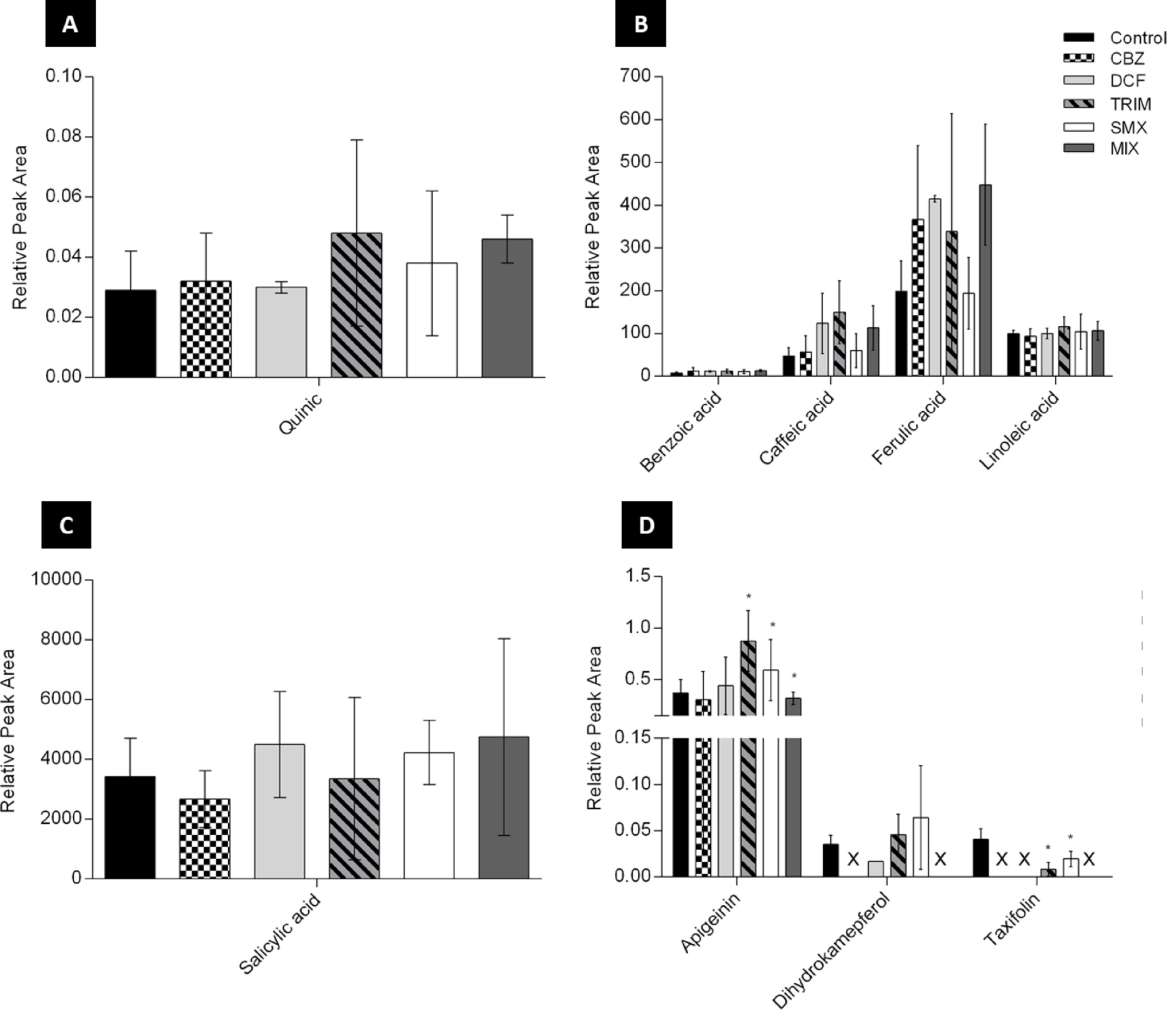
Relative peak area of quinic, benzoic, caffeic, ferulic, linoleic, and salicylic acids, along with apigenin, dihydrokaempferol, and taxifolin, identified in *L. minor* after 5 days of incubation with 5ppb of SMX, TRIM, DCF, CBZ, and their mixture, as well as the control group; statistical differences (p-value ≤ 0.05) against the respective control group are labelled with an asterisk “*”; “X” means that the targeted compound was not detected

In the MIX treatment, 4-aminobenzoic acid and glutamic acid were significantly down-modulated (p-value ≤ 0.05), while the lignin biosynthetic pathway was upregulated, as evidenced by a significant increase in *p*-coumaric acid levels (Fig. 2A, p-value ≤ 0.05), alongside upward trends in caffeic and ferulic acids (Fig. 3B).

### The effect of SMX, TRIM, DCF, CBZ, and MIX on the flavonoid pathway

SMX treatment increased naringenin content (Table S3), the general precursor of all flavonoids, leading to higher apigenin (1.6-fold, p-value ≤ 0.05) and dihydrokaempferol (2-fold) content compared to control (Fig. 3D). This pattern suggests preferential flux towards flavone and flavonol biosynthesis rather than anthocyanin formation. However, the taxifolin turned out to be down-modulated and considered the anthocyanin precursor (Fig. 3D).

Upregulation of *trans-*cinnamic 3.9-fold (*p*-value ≤ 0.05) and *p*-coumaric acids 2.2-fold (Fig. 2A) further confirms activation of the phenylpropanoid pathway. Additionally, *o*-coumaric acid levels tended to increase 2.6-fold compared with the control extracts (Fig. 2A), suggesting that *Lemna* may have used it as a precursor for salicylic acid synthesis (Mishra & Baek, 2021).

TRIM strongly activated the phenylpropanoid pathway, as shown by increased phenylalanine and trans-cinnamic acid levels (*p*-value ≤ 0.05), likely due to enhanced phenylalanine ammonia-lyase activity. Caffeic and ferulic acids were higher under TRIM than SMX exposure (up to 3.2- and 2.2-fold, respectively), while tyrosine levels were similar in both treatments, suggesting up-regulation of the lignin pathway. In addition, flavonoid biosynthesis was stimulated, with increased naringenin (Table S3) and apigenin (2.4-fold vs. control; *p* ≤ 0.05), whereas taxifolin decreased (Fig. 3D). Overall, TRIM shifted the phenylpropanoid pathway towards flavonoid and lignin biosynthesis rather than anthocyanin production.

However, *Lemna* showed upregulation of the phenylpropanoid pathway upon exposure to higher DCF doses (3 and 30 mg/L), as previously reported (Wahman et al., 2022), suggesting a stress response.

CBZ data showed a down-modulation in flavonoids and anthocyanin pathways due to the dihydrokaempferol and taxifolin down-modulation (Fig. 3D). Moreover, naringenin was similar to the control (< LOD; Table S3).

In the MIX treatment, flavonoids and anthocyanin pathways were down-modulated (Figs. 2 and 3), mostly due to DCF and CBZ effects on *L. minor*. Uptake data (Fig. S4) showed reduced accumulation of SMX and TRIM in the MIX treatments compared with single-drug treatment. Moreover, DCF was not detected in the *Lemna* MIX extracts, suggesting the presence of interaction effects. Overall, *L. minor* effectively responded metabolically to pharmaceutical stress, supporting its use as an in situ biomonitor.

Liu and co-workers (2013) reported increased expression of the flavanone 3-hydroxylase (F3H) gene, a key enzyme in the flavonoid biosynthetic pathway, under UV-B radiation and drought stress in *Reaumuria soongorica*. Additionally, the phenylpropanoid scavenges ROS and enhances the plant’s resistance to abiotic stress (Sharma et al., 2019). Thus, the accumulation of phenolic compounds could be due to the up-regulation of the key biosynthetic enzymes, including phenylalanine ammonia-lyase, chalcone synthase, shikimate dehydrogenase, cinnamyl alcohol dehydrogenase, and polyphenol oxidase.

### The effect of SMX, TRIM, DCF, CBZ, and MIX on the lignin pathway

Lignin is a key plant defence polymer that contributes to antimicrobial resistance, structural support, water transport, and the formation of a physical barrier against stress (Xie et al., 2018). As reported, lignin accumulation can be regulated by plant pathogen-induced activation of quinate/shikimate p-hydroxycinnamoyltransferase, with cinnamate 4-hydroxylase and caffeic acid O-methyltransferase acting as important downstream enzymes in the pathway (Xie et al., 2018).

In the present study, benzoic acid (1.5-fold) and salicylic acid (1.3-fold) showed similar levels in DCF- and TRIM-treatments, likely due to the reduced availability of o-coumaric acid (Fig. 3B&C). In contrast, TRIM exposure led to marked increases in caffeic acid (2.6-fold) and ferulic acid (2.8-fold) (Fig. 3B), together with a significant rise in tyrosine levels (p-value ≤ 0.05). These changes suggest an overall up-regulation of the lignin biosynthetic pathway under TRIM treatment.

A similar increase in lignin-related metabolites was observed in CBZ treatment. However, salicylic acid was down-modulated, despite the up-regulation of o-coumaric acid, indicating that the observed increase in *o*-coumaric acid (< 2-fold) was insufficient to stimulate salicylic acid synthesis (Fig. 2A). Overall, lignin accumulation in *Lemna* exposed to TRIM may be associated with the enhanced activity of key enzymes involved in the lignin biosynthetic pathway.

### The effect of SMX, TRIM, DCF, CBZ, and their mixture on lemna fatty acid contents

Linoleic acid was quantified to assess the impact of the different pharmaceutical exposures on *Lemna’s* fatty acid content. Linoleic acid concentrations followed the order: TRIM (396.5) > MIX (106.7) > SMX (104.5) > DCF (100.2) > control (99.5) > CBZ (94.3) (Fig. 3B, Table S3). Compared with the control, linoleic acid was up-modulated in all treatments except CBZ, indicating that SMX, TRIM, and DCF enhance the fatty acid production in *Lemna*.

Further, DCF at higher concentrations activated arachidonic acid metabolism and the biosynthesis of unsaturated fatty acids. This was supported by the untargeted analysis, which showed an increase in 9,12-octadecadienoic acid (linoleic acid), confirming the enhancement of arachidonic acid metabolism. Arachidonic acid is also considered a potent elicitor of programmed cell death and defence responses and induces resistance to viruses by producing plant oxylipins (Savchenko et al., 2010); this shift suggests that DCF exposure induces a stress-related defence mechanism in *L. minor*.

## Conclusion

Due to the pharmaceutical’s stress effect, the plant metabolomics approach is used to investigate specific metabolic pathway changes. Evaluating metabolites in the specific pathway indicates changes within it. The phenylalanine, tyrosine, tryptophan biosynthesis, and phenylpropanoid pathways were selected based on previous results. The response of *Lemna* was specific to each drug. SMX and TRIM blocked the folate biosynthesis pathway in *Lemna minor*, as in bacteria. The 4-aminobenzoic acid seems to accumulate in *Lemna* incubated with SMX, TRIM, DCF, CBZ, and MIX. SMX, TRIM, and DCF up-regulated the flavonoid pathway in incubated *Lemna*. Upon DCF treatment, naringenin and apigenin concentrations were approximately equal and higher than those of the control, respectively. Moreover, the concentrations of dihydrokaempferol and taxifolin were lower than those in the control extracts. The presence of pharmaceuticals such as DCF and CBZ in aquatic environments can affect the biosynthetic pathways of *L. minor*. These responses support the use of *L. minor* as a suitable model to study the pharmaceutical-induced stress response on plant metabolism and associated pathways.

The phenylalanine, tyrosine, tryptophan biosynthesis, and phenylpropanoid pathways can be used to characterise the presence of the SMX, TRIM, DCF, CBZ, or their mixture in the water. The folate biosynthetic pathway can serve as an indicator of SMX-contaminated water. The change in concentration of metabolites could be an indicator for each pharmaceutical. However, future research should focus on the genetic level to further elucidate the mechanisms by which different pharmaceuticals affect *Lemna* pathways.

## Supplementary Information

Below is the link to the electronic supplementary material.

Supplementary Material 1

## Data Availability

The datasets generated during and/or analysed during the current study are available from the corresponding author upon reasonable request.
