# Supplementary material for: Impact of sulfamethoxazole, trimethoprim, diclofenac, carbamazepine, and their mixture on the metabolism of Lemna minor: a targeted metabonomic study
Source: Metabolomics. 2026 Mar 7;22(2):33. doi: 10.1007/s11306-026-02405-9 (PMC12967537; doi:10.1007/s11306-026-02405-9)
Supplement: Supplementary file 1 — Supplementary Material 1 [file 11306_2026_2405_MOESM1_ESM.docx]

**Impact of sulfamethoxazole, trimethoprim, diclofenac, carbamazepine, and their mixture on the metabolism of *Lemna minor*: a targeted metabonomic study**

Rofida Wahman^1,2,3,a,*^, Peter Schröder^4,b^, Geoffroy Duporté ^3^, Serge Chiron^3^, Jörg Drewes^2 ,^ [Andrés Sauvêtre](http://frontiersin.org/people/u/197112)^3,#^, and Catarina Cruzeiro^4,c,d,#^

^1^Chair of Urban Water Systems Engineering, Technical University of Munich, Am Coulombwall 3, 85748 Garching, Germany.

^2^Pharmacognosy Department, Faculty of Pharmacy, Assiut University, Assiut, Arab Republic of Egypt/Egypt.

^3^HydroSciences Montpellier, IRD, CNRS, University of Montpellier, Montpellier, France.

^4^German Research Center for Environmental Health, Unit Environmental Simulation, Helmholtz Zentrum München, Ingolstädter Street 1, 85764 Neuherberg, Germany.

***Corresponding authors**: Rofida.wahman@tum.de

# Both authors contributed equally to this work; joint authors

Present address:

^a^ Pharmacognosy Department, Faculty of Pharmacy, Assiut University, Assiut, Arab Republic of Egypt/Egypt

^b^ Chair of Organic Agriculture and Agronomy, TUM School of Life Sciences Weihenstephan, Technical University of Munich, Liesel-Beckmann-Str. 2, 85354, Freising, Germany.

^c^ LSRE-LCM – Laboratory of Separation and Reaction Engineering - Laboratory of Catalysis and Materials, Faculty of Engineering, University of Porto, Rua Dr. Roberto Frias, 4200-465 Porto, Portugal

^d^ ALiCE – Associate Laboratory in Chemical Engineering, Faculty of Engineering, University of Porto, Rua Dr. Roberto Frias, 4200-465 Porto, Portugal


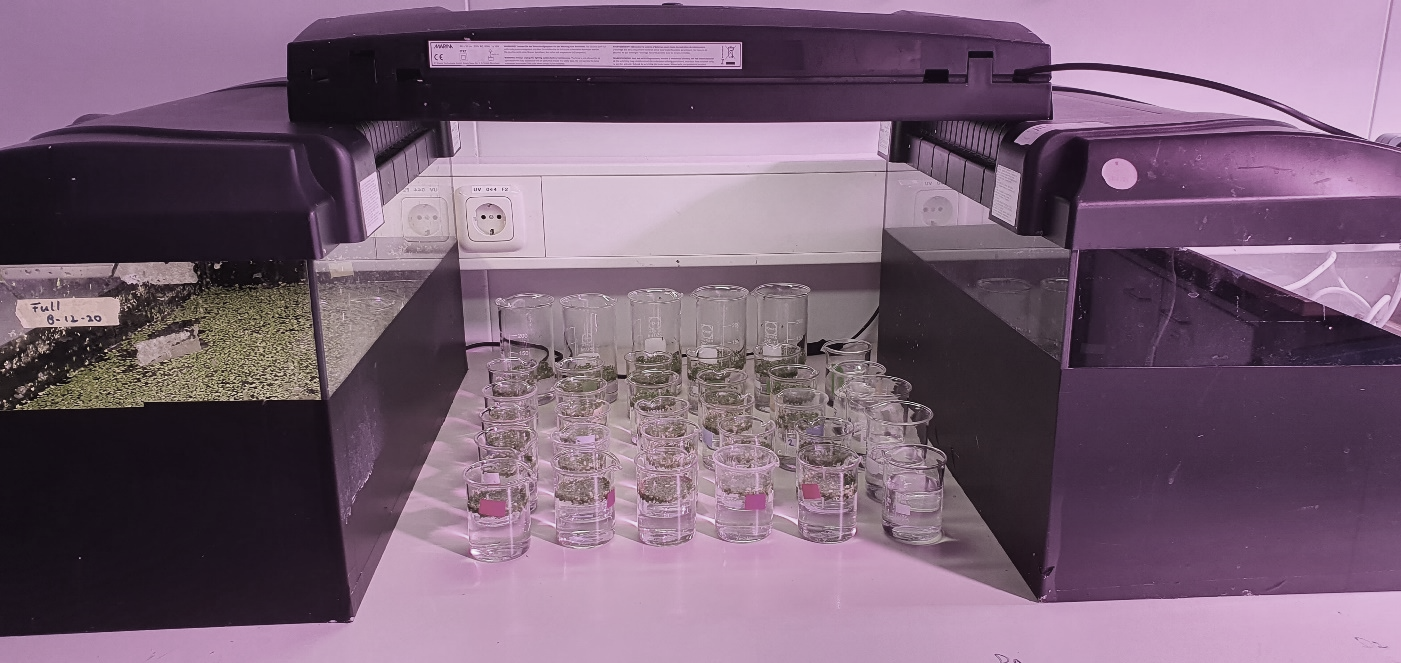


Fig. S1. Photo of Lemna minor exposed to SMX, TRIM, DCF, CBZ, and a mixture of them (MIX) at 5ppb (5µg/L) in Steinberg media for 5 days.


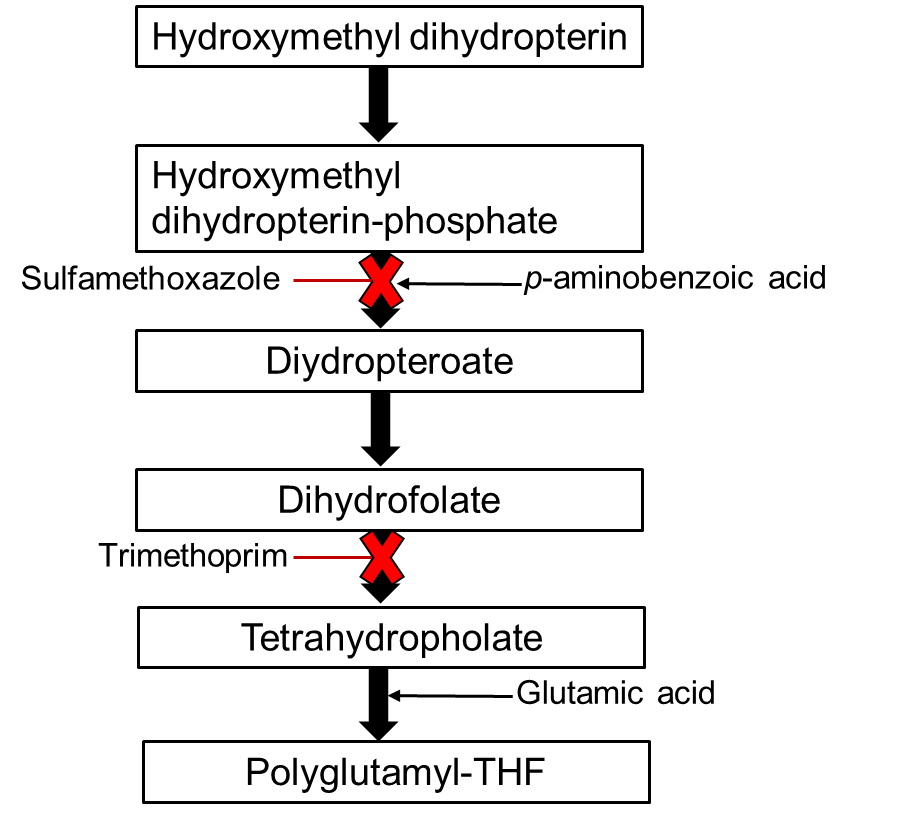


Fig S2. The folate pathway in the mitochondria of a plant cell when is suspected to be inhibited by SMX and TRIM. SMX targets dihydropteroate synthase (DHPS), and trimethoprim targets dihydrofolate reductase (DHFR) in the folate biosynthetic pathway.

Table S1: Steinberg solution composition.

| **Steinberg solution** | **Reagents** | **Molarity (mol/L)** | **Solution used (mL/L)** |
| --- | --- | --- | --- |
| Solution 1 | KNO_3_ | 1.74E+02 | 2 |
|  | KH_2_PO_4_ | 3.32E+01 |  |
|  | K_2_HPO_4_ | 3.59E+00 |  |
| Solution 2 | MgSO_4_.7H_2_O | 2.04E+01 | 2 |
| Solution 3 | Ca(NO_3_)_2_.4H_2_O | 6.20E-01 |  |
| Solution 4 | H_3_BO_3_ | 1.90E-03 | 1 |
|  | ZnSO_4_.7H_2_O | 6.00E-04 |  |
|  | Na_2_MOO_4_.2H_2_O | 2.00E-04 |  |
|  | MnCl_2_.4H_2_O | 9.00E-04 |  |
| Solution 5 | FeCl_3_.6H_2_O | 2.81E+00 | 1 |
|  | Na_2_.EDTA.2H_2_O | 4.03E+00 |  |

| Name | Mean RT (Min.) | SD | %RSD | Molecular ion species |
| --- | --- | --- | --- | --- |
| Carbamazepine-d10 | 12.84 | 0.01 | 0.09 | [M+H]^+^ |
| Sulfamethoxazole-d4 | 11.96 | 0.02 | 0.15 | [M+H]^+^ |
| Diclofenac-d4 | 15.06 | 0.07 | 0.49 | [M+H]^+^ |
| Trimethoprim-d3 | 16.04 | 0.01 | 0.08 | [M+H]^+^ |
| Ibuprofen | 15.90 | 0.10 | 0.66 | [M-H]^-^ |

Table S2. The average retention time (RT, min), standard deviation (SD), and relative standard deviation (%RSD) of the listed internal standards (n=30 injections).

Fig S3. Mass spectrum (m/z) of (a) phenylpyruvate; (b) o-coumaric acid, (c) p-coumaric acid.


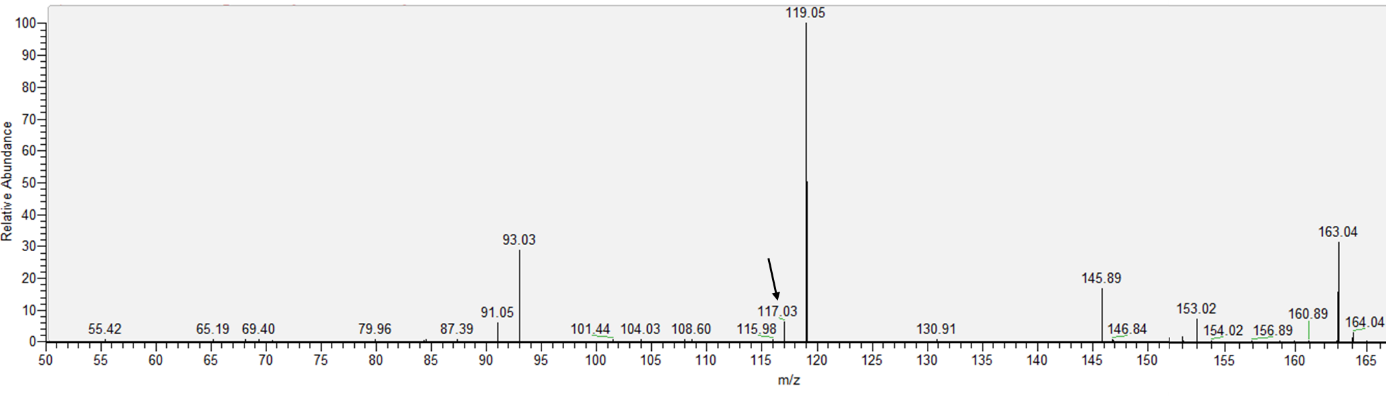

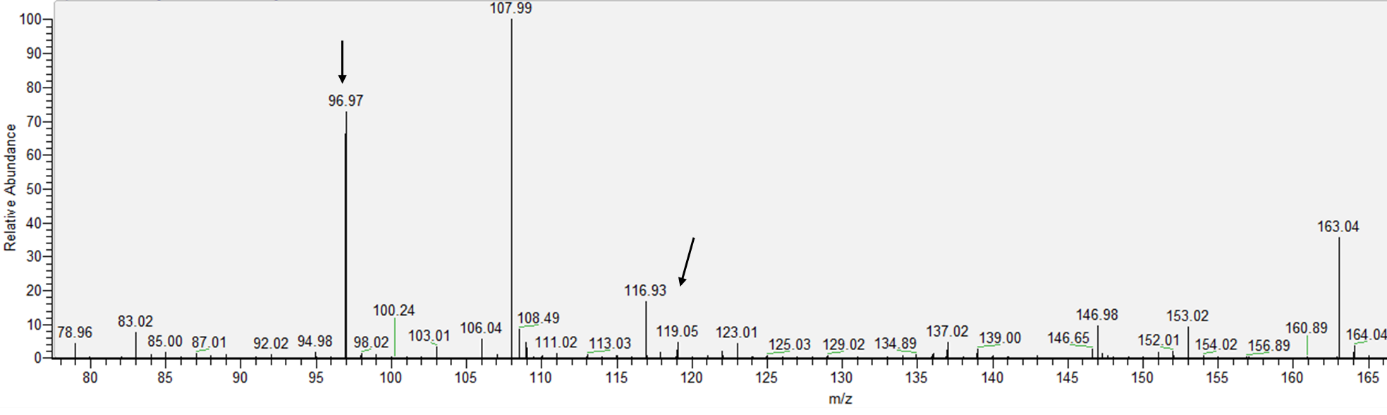

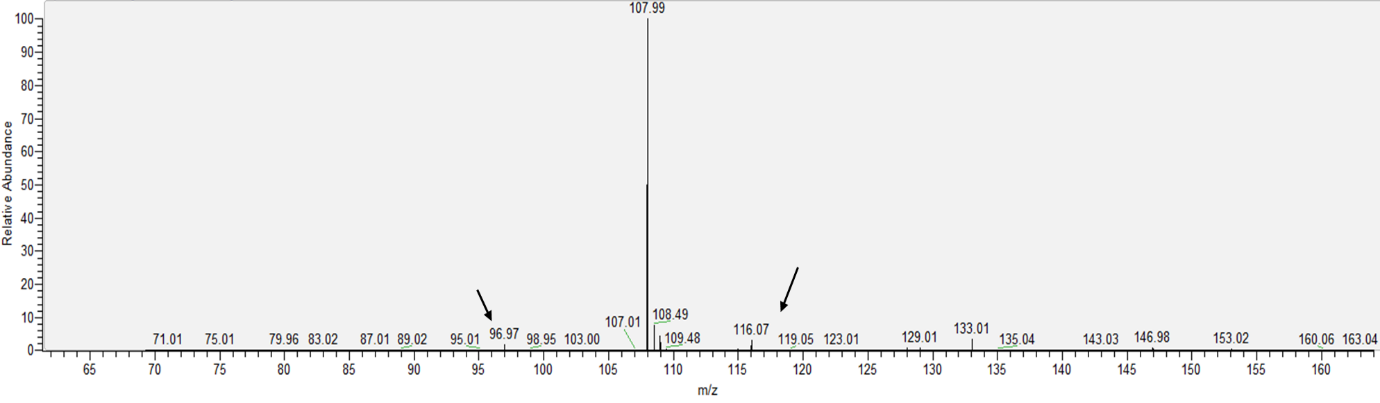


b

a

c

### *
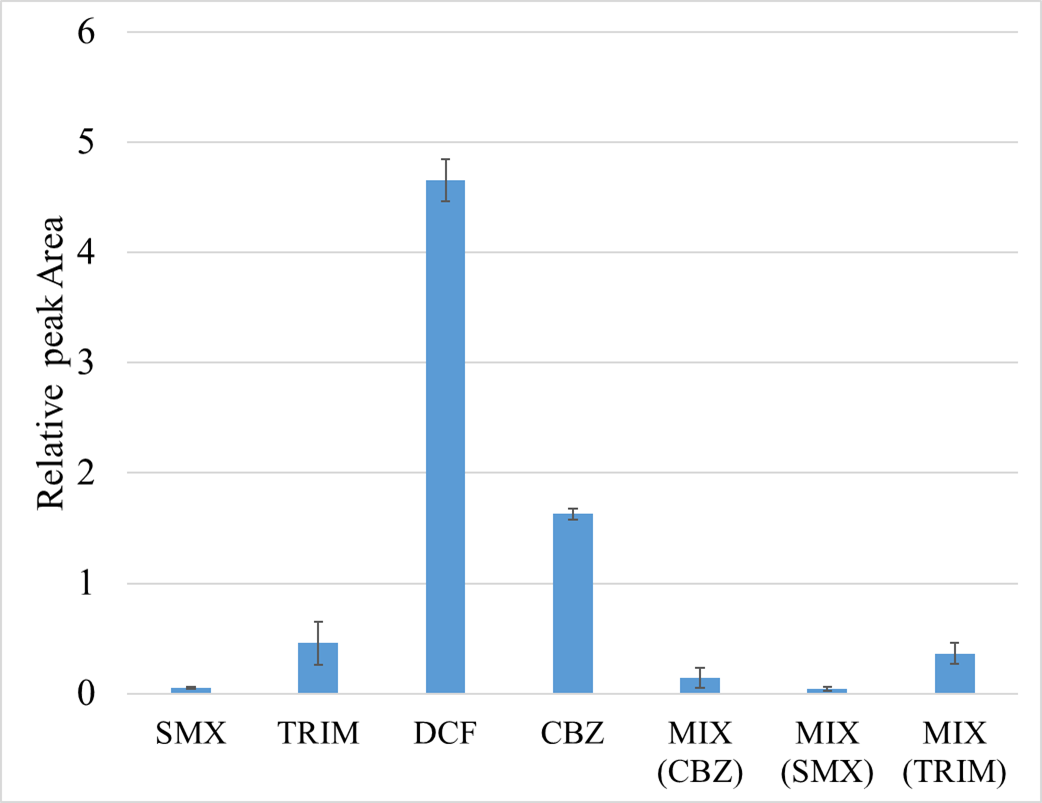
*

Fig S4. The relative peak areas of SMX, TRIM, DCF, CBZ, and their mixture in the Lemna minor extracts.

Table S3. The mean and standard deviation of different metabolite intensities after normalisation against carbamazepine-d10, assigned to positive mode.

---: under the limit of detection

|  | SMX |  | TRIM |  | Control | | DCF | | CBZ |  | MIX |  |
| --- | --- | --- | --- | --- | --- | --- | --- | --- | --- | --- | --- | --- |
|  | Mean | SD | Mean | SD | Mean | SD | Mean | SD | Mean | SD | Mean | SD |
| Apigenin | 0.5917 | 0.2528 | 0.8765 | 0.2954 | 0.3692 | 0.1309 | 0.4397 | 0.2803 | 0.3040 | 0.1080 | 0.3175 | 0.0616 |
| Shikimic acid | 2.5652 | 0.7681 | 2.7771 | 1.0869 | 3.3678 | 1.4476 | 3.1657 | 0.7113 | 4.1322 | 0.4738 | 4.2213 | 0.7641 |
| Tyrosine | 2.8561 | 1.3330 | 3.1087 | 1.1121 | 0.6676 | 0.1807 | 3.7038 | 0.5566 | 2.6701 | 0.5698 | 3.3604 | 0.6119 |
| Cinnamic acid | 1.6047 | 0.5025 | 1.9027 | 0.6778 | 0.5960 | 0.1797 | 1.5872 | 0.3764 | 1.3406 | 0.5681 | 2.5454 | 0.4454 |
| Linoleic acid | 104.5389 | 41.1481 | 396.5225 | 24.3421 | 99.5106 | 8.9559 | 100.2405 | 11.8920 | 94.3306 | 17.5218 | 106.7070 | 21.7614 |
| Ferulic acid | 123.7868 | 84.2098 | 270.8483 | 14.8380 | 122.9662 | 71.3676 | 338.6366 | 7.4107 | 299.8371 | 28.4161 | 361.6101 | 82.4137 |
| Salicylic acid | 2840.6425 | 1076.4631 | 3357.7723 | 1648.2629 | 2906.5876 | 793.0722 | 3762.0003 | 1596.5627 | 2680.9110 | 948.1404 | 3985.8334 | 579.4829 |
| Chrosimic/Prephenic acid | 12.6074 | 5.9244 | 14.7971 | 6.3606 | 8.9646 | 4.3588 | 15.8986 | 6.6956 | 13.6041 | 7.7233 | 19.6693 | 5.7315 |
| *p*-Coumaric acid | 35.3314 | 19.4901 | 38.3379 | 17.0957 | 27.7534 | 11.9311 | 66.4549 | 14.3068 | 50.4442 | 21.2574 | 87.3169 | 30.3848 |
| *o*-Coumaric acid | 20.5143 | 9.7127 | 10.0605 | 4.4833 | 4.5263 | 1.3913 | 14.8046 | 2.5898 | 10.5267 | 4.6241 | 22.6751 | 17.4022 |
| Phenylpyruvate | 28.1967 | 13.2444 | 42.7783 | 19.4982 | 16.1577 | 5.0991 | 38.6908 | 12.3294 | 29.2142 | 20.9294 | 49.7198 | 14.1618 |
| Benzoic acid | 10.5448 | 4.6233 | 12.6783 | 4.6610 | 7.9039 | 2.5789 | 11.7409 | 1.5536 | 12.6239 | 7.5266 | 13.3536 | 2.4773 |
| 5-hydroxy phenyl pyruvate | 83.7191 | 49.1668 | 77.9732 | 36.7072 | 42.8652 | 7.0837 | 143.7973 | 26.2147 | 96.0870 | 42.6248 | 147.2426 | 69.1958 |
| Caffeic acid | 60.0967 | 40.2215 | 149.7107 | 73.2774 | 47.2659 | 19.6823 | 123.6932 | 70.5740 | 101.7882 | 41.4965 | 113.5663 | 52.0986 |
| Naringenin | 0.0852 | 0.0145 | 0.0983 | 0.0474 | --- | --- | --- | --- | --- | --- | --- | --- |

Table S4. The mean and standard deviation of different metabolite intensities after normalisation against Ibuprofen, assigned to negative mode.

|  | SMX | | TRIM | | Control | | DCF | | CBZ | | MIX | |
| --- | --- | --- | --- | --- | --- | --- | --- | --- | --- | --- | --- | --- |
|  | Mean | SD | Mean | SD | Mean | SD | Mean | SD | Mean | SD | Mean | SD |
| Phenylalanine | 117.6525 | 24.6398 | 91.3023 | 48.3821 | 23.3408 | 7.8579 | 118.9190 | 37.6988 | 147.7161 | 11.0249 | 52.7724 | 24.2711 |
| Tryptophan | 415.5987 | 62.1477 | 393.1164 | 167.7497 | 97.6274 | 26.0372 | 401.7505 | 21.6777 | 283.2876 | 99.6746 | 269.1168 | 153.7202 |
| Glutmatic acid | 38.3397 | 5.2660 | 39.7024 | 11.8161 | 24.0494 | 4.4507 | 30.5486 | 11.5110 | 55.4927 | 6.3681 | 23.4120 | 13.4980 |
| Anthranilic acid | 0.0085 | 0.0026 | 0.0041 | 0.0017 | 0.0035 | 0.0014 | 0.0071 | 0.0039 | 0.0099 | 0.0013 | 0.0038 | 0.0007 |
| 4-Amino benzoic acid | 0.1314 | 0.0211 | 0.1253 | 0.0503 | 0.0697 | 0.0414 | 0.1091 | 0.0205 | 0.1798 | 0.0472 | 0.0527 | 0.0431 |
| Indole | 0.0634 | 0.0034 | 0.0077 | 0.0037 | 0.0019 | 0.0005 | 0.0120 | 0.0030 | 0.0084 | 0.0020 | 0.0045 | 0.0017 |
| Apigenin | 0.0179 | 0.0057 | 0.0288 | 0.0099 | 0.0051 | 0.0003 | 0.0141 | 0.0002 | 0.0050 | 0.0024 | 0.0030 | 0.0011 |
